# Mammalian Cell Spheroids on Mixed Organic–Inorganic Superhydrophobic Coating

**DOI:** 10.3390/molecules27041247

**Published:** 2022-02-12

**Authors:** Michele Ferrari, Francesca Cirisano, M. Carmen Morán

**Affiliations:** 1 CNR-ICMATE Istituto di Chimica della Materia Condensata e di Tecnologie per l’Energia, via De Marini, 6, 16149 Genova, Italy; francesca.cirisano@ge.icmate.cnr.it; 2 Institut de Nanociència i Nanotecnologia—IN2UB, Universitat de Barcelona, Avda. Diagonal, 645, 08028 Barcelona, Spain; 3 Secció de Fisiologia, Departament de Bioquímica i Fisiologia, Facultat de Farmàcia i Ciències de l’Alimentació, Universitat de Barcelona, Avda. Joan XXIII, 27-31, 08028 Barcelona, Spain

**Keywords:** agarose, 3D profilometry, circularity, mammalian cells, size distribution, spheroids, superhydrophobic

## Abstract

Three-dimensional cell culture has become a reliable method for reproducing in vitro cellular growth in more realistic physiological conditions. The surface hydrophobicity strongly influences the promotion of cell aggregate formation. In particular, for spheroid formation, highly water-repellent coatings seem to be required for the significant effects of the process. In this work, surfaces at different wettability have been compared to observe their influence on the growth and promotion of aggregates of representative mammalian cell lines, both tumoral and non-tumoral (3T3, HaCat and MCF-7 cell lines). The effect of increased hydrophobicity from TCPS to agarose hydrogel to mixed organic–inorganic superhydrophobic (SH) coating has been investigated by optical and fluorescence microscopy, and by 3D confocal profilometry, in a time scale of 24 h. The results show the role of less wettable substrates in inducing the formation of spheroid-like cell aggregates at a higher degree of sphericity for the studied cell lines.

## 1. Introduction

Adhesion control of cells to surfaces has an essential role in designing materials for biomedical applications, determining substrates that affect many aspects of cell function, such as spreading, migration, proliferation, and differentiation. Surface chemistry and topography influence cell behavior, and the response of cells to the surface has been studied as functions of the cell size and surface features [1]. In fact, surface roughness (Sa) modulates the biological response of tissues in contact with the substrate [2]. Cells have been proven to be influenced by both isotropic and anisotropic surfaces, while strong differences have been observed as a function of the cell type, and some preferences for disordered systems, with selective responses for surface chemical modifications, correlated to changes occurring at the micro/nano scale [3]. 

Wettability behavior is another important aspect that can influence cell adhesion and growth. A surface can be defined as hydro/oleo-phylic when the water or oil contact angle (CA) is <90°, hydro/oleo-phobic when CA > 90°, and superhydro/oleo-phobic (SH) when CA > 150°. Amphiphobicity is a feature of a surface that shows both oil and water repellence. In particular, SH surfaces (SHS) result from a combination of low surface energy material with a specific surface morphology (micro/nanoroughness) [4].

Culturing cells in three dimensions has become a challenging model for reproducing in vitro cellular growth in more realistic physiological conditions. Three-dimensional cell aggregates or spheroids are interesting for maintaining cellular electrical activity and intracellular functions [5,6], and, for this reason, spheroid culture can be regarded as a promising method to provide novel insights into drug screening in vitro [7]. 

The physical properties of the tumor cell environment have been discussed in [8], with the aim of explaining the cause–effect relationship of physico-mechanical signals in cancer formation. Usually, non-adhesive coatings or substrates at particular wettability promote the formation of 3D cellular spheroids, where cell clustering occurs in environments under gravity or shear stress. Under these conditions, intercellular interaction mechanisms are activated, with the expression of molecules such as E-cadherin for compact cellular structure and the inhibition of caspase-based cell death [9,10].

Despite the recent developments observed in this field [11], to date, the literature still provides a limited number of records related to highly water- and/or oil-repellent materials, such as superhydrophobic, oleophobic, amphiphobic, etc., to be used in cell biology and, in particular, devoted to specifically promoting the formation of spheroids or three-dimensional aggregates. For example, tunable water repellence has been exploited by various authors to produce platforms for growing spheroids independently from surface interactions with the substrates. In these methods, cells aggregate in a confined liquid volume, hanging drop or entrapped in small, superhydrophobized wells [12,13,14].

Nevertheless, when surface specificity is under investigation, the development of coatings and materials at high hydrophobicity opens more flexible applications, due to the combination of surface chemistry and morphology. In this direction, some authors in the literature have studied superhydrophobic, or even superamphiphobic, substrates to develop spheroids [15,16,17]. Authors have also developed a robust platform using metallic mesh as an omniphobic, simple and reusable surface for the high-performance culture of multicellular and heterogeneous multicell-type spheroids. A hierarchical, textured aluminum mesh was silanized, providing an inert, low wettable surface for the long-term culture of spheroids [18]. To avoid ligand coating in the substrate, a plasma-treated, silicone-based material was prepared to promote and modulate cell adhesion undergoing mechanical surface stretching [19].

The effect of the physico-mechanical properties of alternative materials for growing cell lines and tissue engineering has been investigated in [20], where the authors compared modulus surface roughness as a function of the deuteration of agarose gel. 

Moreover, spheroid characterization can still be considered a work in progress, in terms of the limitations of analytical tools, since most of the available techniques were developed and standardized for 2D models, under the assumption of spherical growth of the aggregates [21,22], to be used in 3D culture. To upgrade this topic, advanced, non-destructive techniques, such as confocal 3D profilometry, have been used and coupled to more traditional techniques by the present authors to evidence different cell evolution states [23].

Although spheroids have been widely used as an in vitro model of tissues and organs in the field of cancer, the extension of this 3D model to non-tumoral cells could be of great interest. Three-dimensional skin equivalents have been developed over the last few decades for studying complex properties of skin, and for drug discovery and clinical applications for skin regeneration in chronic wounds. However, very few studies have reported representative skin cells as spheroids [24].

Previous works in our lab have demonstrated that hydrophobicity/superhydrophobicity can modulate cell adhesion and spreading [25,26,27]. In the present study, however, the direct effect of superhydrophobicity on spheroid-like structures has been evaluated and compared with agarose-derived hydrogels, a positive control in promoting spheroids, and TCPS, as a standard cell culture material, was considered a positive control for the 2D cell culturing. In this work, the promotion of 3D spheroid-like cell cultures has been studied as a function of surfaces at different wettability, from hydrophilic to superhydrophobic. Furthermore, both tumoral and non-tumoral cell lines have been grown in the proposed substrates, in order to compare different surface parameters and analysis techniques. The role of surface properties in the differentiation of cell-size populations, as a function of growth dynamics, has been evidenced.

## 2. Results and Discussion

### 2.1. Physicochemical Characterization of the Surfaces

In this work, surfaces with different wettability properties have been used. Commercial TCPS plates constitute the control surface, with excellent cell adhesion efficiency [28]. In addition, agarose-derived hydrogel (0.4% *w*/*v*) was considered a putative substrate to induce spheroid formation [29]. 

The mixed organic–inorganic-coated glass was prepared on glass by the spray coating technique, using the dispersion of fumed silica nanoparticles at a concentration of 2 g/L in a fluoropolymer blend [30]. Table 1 summarizes the physicochemical characteristics of the proposed substrates, ranging from hydrophilic to superhydrophobic, based on the CA values. 

Agarose gel contains polysaccharide molecules with a sugar ring and hydrophilic −OH groups located on both sides. Independently, upon molecular orientation and conformation at the interface, a certain number of −OH groups will be at the gel–air interface, conferring a more wettable substrate to agar gel, with a lower contact angle with water and higher contact angle hysteresis (CAH). On the other side, the presence of fluorine-substituted hydrocarbon chains on SHS, in combination with the hierarchical roughness provided by silica, offers a highly water-repellent substrate, with high CA and lower CAH. 

**Figure 1:**
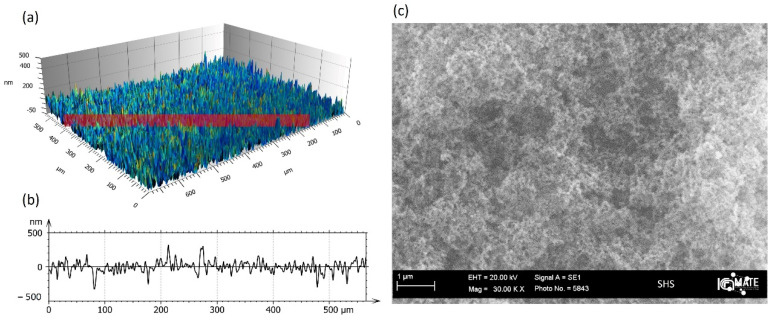
Three-dimensional image of SH sample (**a**) and correlated roughness profiles (**b**) acquired by 3D interferometric and confocal profilometer Sensofar S-Neox: Sa = 70 ± 2 nm. Representative SEM image of SH sample microstructure at 30 Kx (**c**), reference bar 2 µm.

Only SHS was experimentally characterized in terms of wettability (CA), morphology and roughness to gain further insights into the relationship between surface geometry and cell material adhesion. The homogeneous distribution of the SH coating on the glass substrate has been observed and confirmed by wettability studies and 3D confocal interferometric profilometry. In order to assess the homogeneous character of the coating deposition, the data about the water contact angle (CA) were collected at at least three different points, and, in each case, CA was over 160°, with drops rolling off the surface. From 3D profilometry, it was observed that the surface showed a topography based on nanometric dual-scaled roughness (Sa), with an average value of 70 nm. Representative 3D images and profiles of the SH coating at 20× are shown in Figure 1a,b, respectively. Figure 1c reports a high-magnification SEM image of SHS, in which it is possible to observe the fine structure of the sprayed coating.

### 2.2. Cell Behavior on 2D Culture and Agarose-Induced 3D Culture

**Figure 2:**
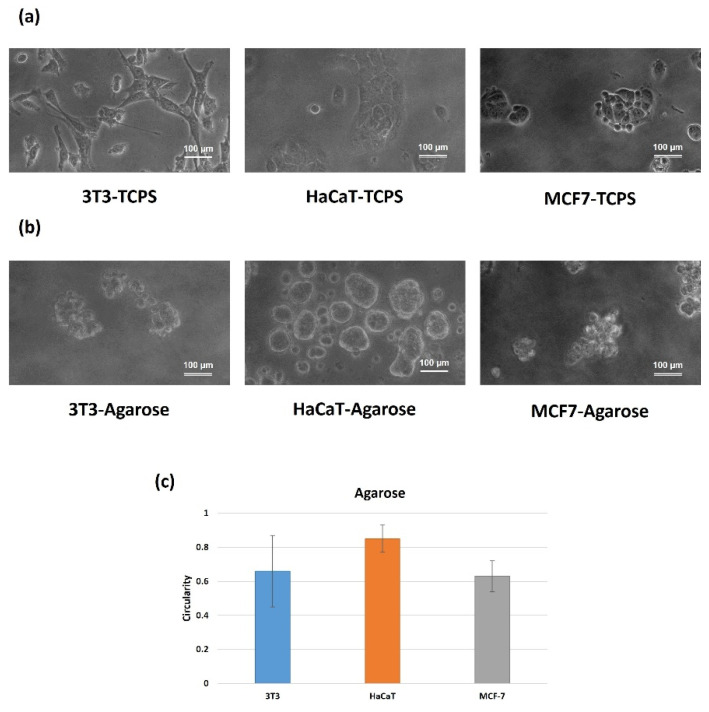
Representative phase-contrast images of cells in 2D culture (**a**), spheroid-like structures (**b**) prepared in 0.4% (*w*/*v*) agarose, and (**c**) measure of the circularity of the agarose-induced structures. Results are expressed as the average of more than 10 single spheroids ± standard deviation. Scale bars represent 100 μm.

Cell culture in TCPS plates was used to assess the morphological characteristics, with controlled 2D features. Figure 2a shows representative images of three cell lines. Accordingly, 3T3 cells show a typical bipolar or multipolar structure, with elongated shapes growing attached to the substrate [31]. Additionally, epithelial-like cells, such as HaCaT and MCF-7 cells, are polygonal in shape, with more regular dimensions and growing attached to the substrate in discrete patches [32]. 

The capability of these cells to form spheroid-like structures was assessed by seeding the corresponding cell lines, at the same cell density, on the top of agarose as a base layer. Hydrogels are recognized to be suitable platforms that promote spheroid formation, due to their ECM-mimicking biophysical properties [33,34,35]. Among the different compounds able to form a hydrogel, agarose is natural, low cost, non-toxic, and cell repellent, promoting spheroid formation. Furthermore, the hydrogels from agarose show appropriate permeability to nutrients and drugs, which is critical for research in the field of spheroid applications [36,37]. 

Using the agarose platform (Figure 2b), the 3D spheroids are typically formed by the cells coming together to form a compact ball, as in the case of the keratinocyte cell line (HaCaT), or grape-like cluster of cells (3T3 and MCF-7 cell lines), due to gravitational pull, with a focus point formed by the meniscus of the agarose [38]. However, despite the non-adherent properties of agarose (CA = 90° and CAH > 10°), the derived hydrogels show some limitations on the cell culture of tumoral cells lacking the activation of specific signaling pathways related to therapeutic processes in tumor cells [39,40].

It is noteworthy that sphericity is an important parameter, as spherical aggregates are more stable, regarding morphological variations throughout growth, and proliferative and necrotic regions [7]. In this work, the geometrical interpretation of spheroid formation has been evaluated, considering the circularity [36,41] of the cross-sectional area, without the common assumption of the spherical symmetry of the ongoing formation of the aggregates [22,23]. Circularity has been calculated as the ratio between the two orthogonal diameters (d_1_ and d_2_) of the spheroid product of the shortest axis and longest axis; circularity = d_1_/d_2_. The circularity values are 1.0 for a perfect circle and values far away to 1.0 for elongated ellipsoids. As shown in Figure 2c, the hydrogel agarose-induced spheroids showing limited circularity values, ranged between 0.6 and 0.8 in all cases, probably induced by a more adhesive substrate, as can be derived from Table 1. 

### 2.3. Cell Behavior on Superhydrophobic Surfaces

As shown in the literature, the high hydrophobicity of the substrate seems to promote the formation of 3D aggregates. A key consideration in the design of in vitro cell culture systems is the cell-to-media volume ratio, which is an important factor that drives cell survival, proliferation and function. Hence, in this work, the spheroid formation on the SH coating has been evaluated as a function of the initial cell density (either 2000 cell/µL or 20,000 cell/µL). 

**Figure 3:**
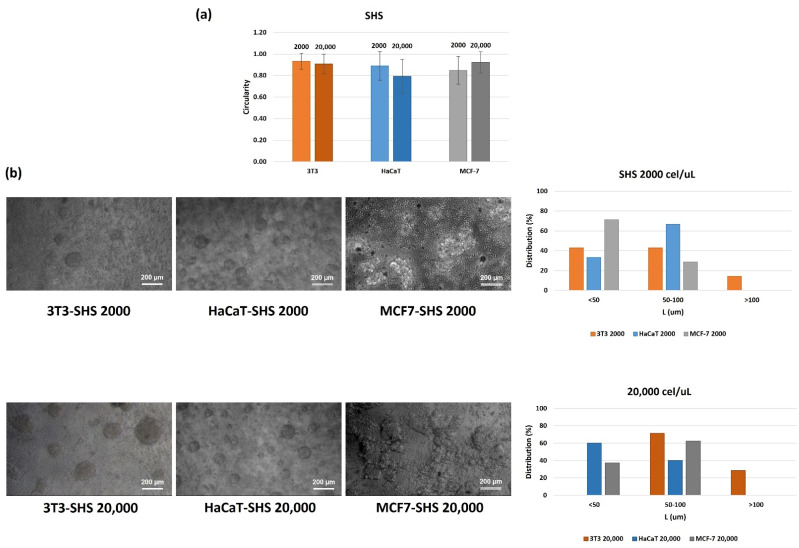
Circularity values (**a**) and representative phase-contrast images and size distribution of spheroids (**b**) as a function of the initial cell density (either 2000 cell/µL or 20,000 cell/µL) after incubation in SH coating for 24 h. Results are expressed as the average of more than 10 single spheroids ± standard deviation. Scale bars represent 200 μm.

Figure 3 shows representative results of the morphological characterization of the SH-induced aggregates. The ratio between the average diameters has been taken as the percentage of circularity achieved by the spheroid in the time window. This cross-sectional circularity seems to be poorly influenced by the concentration in Figure 3a. The circularity varies slightly from the initial cell density, with values ranging between 0.91 and 0.93 for 3T3, 0.90 and 0.8 for HaCaT, and 0.9 and 0.92 for MCF-7, at lower and high cell densities, respectively. The circularity data demonstrated that spheroid formation appears to be promoted by SHS, with more adhesive properties observed compared to the reference substrates (TCPS and agarose).

Considering the circularity values, size has been expressed using only one dimension (d_1_ values). Figure 3b shows representative phase-contrast images and the size distribution population of each cell line. This representation provides further insights into spheroid development dynamics, which could be a characteristic parameter for studying a specific line during the building process of the 3D aggregates. In this case, already at shorter times in comparison with the literature, the size distribution shows significant differentiation, enhanced by the SH coating. The spheroids’ shaping also undergoes possible differentiation as a function of the cell line. The results demonstrated that the spheroid distribution depends on both the cell density and cell line. Thus, in the case of the 3T3 cell line, the lower cell density promotes the formation of spheroids in three different ranges. However, spheroids with sizes <50 and 50–100 µm represent more than 40% of the population, while spheroids > 100 µm represent only 15%. As described in the literature [7,12,13], the size of the aggregates appears to be dependent on the concentration (sometimes it is also a growth limitation factor). In this work, the increase in the initial concentration, by 10 times, promotes the formation of spheroids of higher dimensions, with distribution sizes ranging from 72% for 50–100 µm to 28% for structures > 100 um. Spheroids at the lower size distribution are absent. 

In the case of the HaCaT and MCF-7 cell lines, the size distribution seems to be restricted at the series <50 and 50–100 µm, in which the final distribution depends on both the cell line and the initial cell density. Accordingly, in the case of the HaCaT cell line, using the lower cell density (2000 cell/µL), the result of the series 50–100 µm is preferred (>65%); however, this distribution showed an inversion when the cell density changed to 20,000 cell/μL, for which spheroids <50 µm are most popular (60%). In the case of the tumoral cell line (MCF-7), the opposite trend is observed, promoting the formation of the lowest series at the lowest cell density (72%), growing up to 50–100 μm (63%) as the initial cell density increases. 

**Figure 4:**
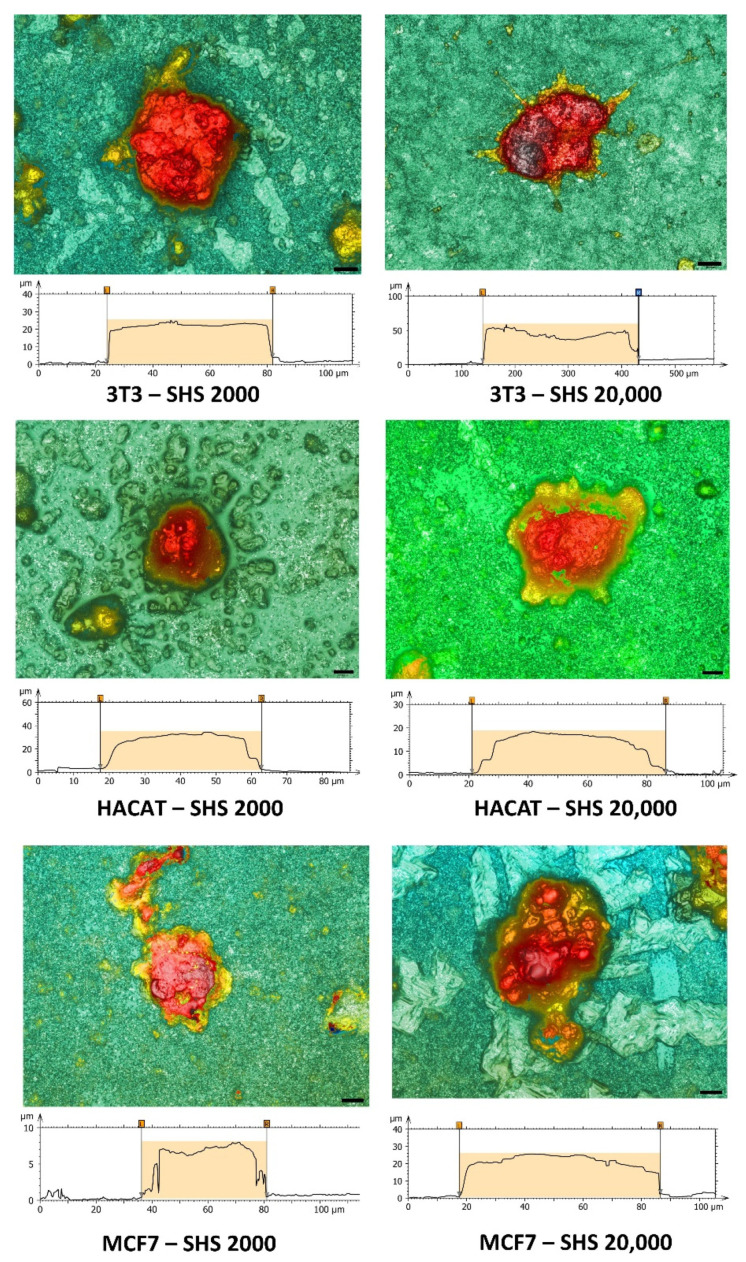
Representative 3D profilometry images in confocal mode (magnification 100×) of spheroids as a function of the initial cell density (either 2000 cell/µL or 20,000 cell/µL) after incubation in SH coating for 24 h, deduced by the corresponding profile sections.

Three-dimensional confocal profilometry images have been used to confirm the size range of the different cell lines. Single aggregate profiles allow the morphology of the cluster and the diameter of the cross-sectional area to be outlined with high accuracy (Figure 4). Three-dimensional confocal profilometry seems to be an effective tool for studying spheroids, providing a simple, non-destructive method to assess surface features with higher accuracy, confirming the data obtained by the other optical methods.

It has been described that spheroid size could determine the cell viability of the 3D model. The mass transportation of nutrients and oxygen within the spheroid could be confined for the larger spheroids, resulting in nutrient depletion and hypoxia in the intimate regions. In addition, based on diffusional limitations, the larger spheroids would show lower proliferation capability [42], and accumulate carbon dioxide and metabolic reaction products as lactate in their inner regions [43]. The effects related to mass transfer limitations may conclude in cell necrosis in the center of the spheroid. Instead, tumor cells on larger spheroids showed higher growth rates than in smaller aggregates [44].

One of the drawbacks of the quantitative characterization of spheroids is the application of techniques and protocols that were initially designed for 2D culturing. When the number of viable cells in spheroids is determined by direct methods, such as manual or automatic counting, the spheroids must be dissociated into individual cells. This process would result in a more or less complicated process as a function of the cell type composing the spheroids. Indirect methods, involving colorimetric and bioluminescent assays, are limited and ineffective, due to the strong cell–cell and matrix–cell interactions, which result in greater exposure to conditions that may cause potential cell damage, affecting cell viability, which is precisely the target parameter.

**Figure 5:**
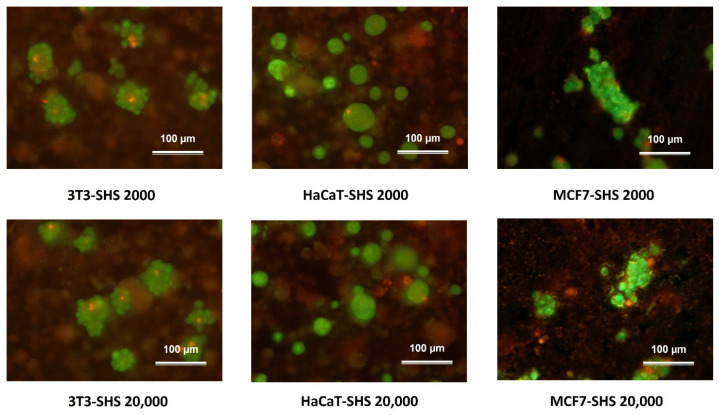
Representative fluorescence microscopy images of spheroids as a function of the initial cell density (either 2000 cell/µL or 20,000 cell/µL) after incubation in the SH coating after 24 h. Green fluorescence indicates AO stain in the live cells and red fluorescence indicates the EtBr stain in the dead cells. Scale bars represent 100 μm.

In this work, acridine orange/ethidium bromide (AO/EB) double staining was used to verify the homogeneous distribution of viable cells in the SH coating-induced spheroids. Figure 5 shows that the viability of the cells during spheroid formation was preserved. The green fluorescence stain in the live cells and red fluorescence stain in the dead cells indicate the well-preserved viability of the cells in the spheroids formed at 24 h of incubation. Live cells emitting green constitute almost the entire spheroid surface, with no or only a few dead cells emitting red. Although different populations were shown, no evident differences in the live/dead distribution were observed. 

The qualitative live/dead assay used in the present work provides valuable information about the real scenario inside the spheroid. Thus, the mass transport of nutrients and oxygen within the spheroid structure may be harmful or beneficial, depending on the desired type of application of the spheroid. For example, for 3D models used as building blocks for tissue engineering, such gradients are detrimental, as they may reduce the applicability of the derived spheroid for the presence of necrotic areas that would affect their assembly of human tissues. However, for 3D tumor models, this characteristic is desirable, mimicking the in vivo tumor microenvironment, which is suitable for studying mechanisms of resistance, migration, tumor invasion, and escape of malignant cells submitted to treatments such as chemotherapy [7]. The results demonstrated that the SH coating was robust enough for spheroid formation with live cells for all the populations.

## 3. Materials and Methods

### 3.1. Materials

Commercially available fluoropolymer blend (Surface Energy 15 mN/m) solution of a fluorosilane polymer (0.1 wt.%), carried in a hydrofluoroether solvent of methoxy-nonafluorobutane (low (320) global warming potential * and zero ozone depletion potential **) was used as received. Fumed silica (EVONIK HDK H15) was purchased from Degussa (Hannover, Germany) with primary particles about 5–30 nanometers in size.

Dulbecco’s modified Eagle medium (DMEM), fetal bovine serum (FBS), L-glutamine solution (200 mM), penicillin–streptomycin solution (10,000 U/mL penicillin and 10 mg/mL streptomycin), phosphate-buffered saline (PBS), d trypsin–EDTA solution (170,000 U/L trypsin and 0.2 g/L EDTA) and Seakem LE Agarose were purchased from Lonza (Verviers, Belgium). The 75 cm^2^ flasks and 24-well cell culture plates were obtained from TPP (Trasadingen, Switzerland). All other reagents were of analytical grade.

### 3.2. Methods

#### 3.2.1. Surface Preparation and Characterization 

Commercial 24-well tissue culture polystyrene (TCPS) plates were used as received or coated with 0.4% (*w*/*v*) of agarose in sterilized Milli-Q water. The coated plate was allowed to solidify completely overnight.

Superhydrophobic surfaces (SHS) were prepared on glass by spray coating technique using the dispersion of fumed silica nanoparticles at a concentration of 2 g/L in fluoropolymer blend. The coating was prepared using a constant distance between surface and nozzle, a pressure of 0.8 bar, and different layer cycles were performed to obtain samples with different surface characteristics. The as-prepared coatings were rinsed in water to assess the high water repellence and remove the eventual residuals that could affect the cell proliferation. 

Surface wettability was investigated by contact angle (CA) by drop shape method using ASTRA view tensiometer [45] allowing real-time drop volume control up to tens of μL for hysteresis studies (advancing/receding CA) and up to 15 frames of frame grabbing. Drops of about 5 μL were deposited onto the uncoated and coated TCPS, and SH-coated glass, and contact angle was measured up to spreading equilibrium. Additionally, in the case of SHS, the surface structure of samples was investigated by 3D confocal and interferometric profilometry (Sensofar S-NEOX, Barcelona, Spain) in order to evaluate the roughness and to acquire the confocal image. The profilometry was chosen to permit a large surface scan, for ease and fast non-destructive use. The profilometry surface characterization was performed according to the standard ISO 25178. Furthermore, SHS microstructure was characterized by scanning electron microscopy (SEM) (LEO 1450VP, LEO ElectronMicroscopy Ltd., Cambridge, UK).

TCPS plates, hydrogel-based agarose and SHS were pre-treated before any cell-involving assays were conducted. Based on Sharma protocol, the pre-treatment consisted of UV sterilizing procedure for 45 min [46].

#### 3.2.2. Cell Cultures

The murine Swiss albino fibroblast (3T3), the immortal human keratinocyte (HaCaT) and the human breast adenocarcinoma (MCF-7) cell lines were grown in DMEM medium (4.5 g/L glucose) supplemented with 10% (*v*/*v*) FBS, 1% (*v*/*v*) L-glutamine and 1% (*v*/*v*) antibiotic at 37 °C and 5% CO_2_. Cells were cultured in 75 cm^2^ culture flasks and were routinely split when cells were approximately 80% confluent.

#### 3.2.3. Conventional 2D and Agarose-Induced 3D Culture

Cells (2 × 10^5^ cells/mL) were seeded into 24-well cell culture plates in the absence or presence of agarose coatings. Then, cells were incubated for 24 h under 5% CO_2_ at 37 °C. Once completed, the cell morphology and growth were monitored by optical microscopy through a Nikon inverted microscope equipped with a video camera (Moticam 1080 HDMI&USB, Moticam 1080 HDMI and USB, Motic Europe, Barcelona, Spain). Images were analyzed with an image processor (Motic Images 3.0 software, Moticam 1080 HDMI and USB (Motic Europe, Barcelona, Spain).

#### 3.2.4. Cell Culture in Superhydrophobic Substrates

Cell behavior into SH substrates was evaluated using SH-coated glasses. In order to ensure that the cell attachment was not affected by the medium culture repellence and sample floating in the Petri dish, cells were seeded into delimited areas of the coated samples fixed by a silicone o-ring.

Cells were seeded at two different cell densities (2 × 10^5^ cells/ mL and 20 × 10^5^ cells/ mL). Cells were incubated for 24 h under 5% CO_2_ at 37 °C. Once completed, the cell morphology and growth were monitored by optical microscopy through a Nikon inverted microscope equipped with a video camera (Moticam 1080 HDMI&USB). Images were analyzed with an image processor (Motic Images 3.0 software).

#### 3.2.5. Profilometry Studies

Three-dimensional confocal and interferometric profilometry S-NEOX (Sensofar, Barcelona, Spain) was used to obtain surface parameters because it allows larger surface scans and ease of use. The surface characterization by profilometry was conducted according to the standard ISO 25178, which provides the rules for the three-dimensional parametric assessment of surface textures.

Cells were seeded at the densities mentioned above into the coated glasses following the standard atmosphere, temperature and time conditions. Then, the spent medium was eliminated, and cells were fixed with 4% (*v*/*v*) paraformaldehyde for 15 min. The fixed cells were maintained in sterile PBS and low temperature (approximately 5 °C) up to the point of being scanned.

The entire surface of the individual o-ring delimitating the areas containing cells was analyzed using confocal mode. Cells on selected areas were chosen, and the corresponding profiles were analyzed with the Sensofar S-NEOX SensoSCAN software (Sensofar, Barcelona, Spain).

#### 3.2.6. Fluorescence Microscopy Studies

To assist the formation of spheroids and the preserved viability of the involved cells, fluorescence microscopy using acridine orange/ethidium bromide (AO/EB) double staining was conducted [47]. Cells were seeded at the densities mentioned above into the coated glasses following the standard atmosphere, temperature and time conditions. First, cells were incubated for 24 h under 5%. Then, the spent medium was eliminated, and the fluorescent dyes AO (0.5 μg/mL) and BE (10 μg/mL) were added. Fluorescence images were acquired with an Olympus BX41 microscope equipped with a a UV-mercury lamp (100 W Ushio Olympus, Olympus Iberia, Barcelona, Spain) and a U-N51004v2-FlTC/TRITC-type filter set (FITC: BP480-495, DM500-545, BA515-535, and TRITC: BP550-570, DM575-, BA590-621). Images were digitized on a computer through a video camera (Olympus digital camera XC50, Olympus Iberia, Barcelona, Spain) and were analyzed with an image processor (Cell-B analysis).

## 4. Conclusions

In this work, the formation of spheroidal aggregates from different cell lines, both tumoral and non-tumoral, was studied in relationship to the hydrophobicity of the substrate. While less hydrophobic substrates also grow three-dimensional aggregates, but with a lower degree of circularity, accordingly with their higher wettability, allowing higher spreading, the superhydrophobicity of the samples coated with a mixed organic–inorganic coating preferentially promotes the formation of spheroids evidencing different dynamics for each cell line, also depending on the cell density. At 24 h of incubation, significant differentiation in size populations could already be observed, allowing, as a future perspective, a promising approach for modeling this kind of phenomena during longer time windows.

Although superhydrophobicity has been used to develop spheroids, most of the examples in the literature provide highly technological solutions. In our case, however, the spray application of an SH coating is suitable for every substrate material, allowing the surface properties to be finely controlled by composition and roughness, via a cheap and easy-to-use method. In this way, we have a more comprehensive range of potential applications to investigate spheroid formation under different conditions of coating composition and surface geometry (roughness).

## Figures and Tables

**Table 1:** Surface characteristics of the substrates.

Substrate Code	Characteristics	Contact Angle (CA)	Contact Angle Hysteresis (CAH)	Surface Roughness (Sa)
TCPS Agarose SHS	Tissue culture polystyrene Agarose-coated TCPS Fluoropolymer blend + silica NPs	66° 80–90° >150°	>10° <5°	1–6 nm [28] 16–18 nm [17] <70 nm

## Data Availability

Not applicable.
